# Neuromuscular Training following Anterior Cruciate Ligament reconstruction – Pain, Function, Strength, Power & Quality of Life Perspective: A Randomized Control Trial

**DOI:** 10.12669/pjms.38.8.5730

**Published:** 2022

**Authors:** Kehkshan Khalid, Naveed Anwar, Ghulam Saqulain, Muhammad Faheem Afzal

**Affiliations:** 1 Kehkshan Khalid, MS SPT. Lecturer, Riphah College of Rehabilitation & Allied Health Sciences, Riphah International University, Lahore, Pakistan; 2 Naveed Anwar, Assistant Professor, Riphah College of Rehabilitation & Allied Health Sciences, Riphah International University, Lahore, Pakistan; 3 Dr. Ghulam Saqulain, F.C.P.S (Otorhinolaryngology). Head of Department & Professor of Otorhinolaryngology, Capital Hospital PGMI, Islamabad, Pakistan; 4 Muhammad Faheem Afzal, MS SPT. Senior Lecturer, Riphah International University, Islamabad, Pakistan

**Keywords:** Anterior cruciate ligament, Effectiveness, Neuromuscular training, Rehabilitation, Strength Training

## Abstract

**Objectives:**

To determine the effectiveness of neuromuscular physical Therapy as compared to strength training following anterior cruciate ligament reconstruction in terms of pain, function, quality of life, strength and power of participants.

**Methods:**

A randomized clinical trial was conducted at Kanaan Physiotherapy & Spine Clinic, Lahore, Pakistan from July 2020 to December 2020. Seventy-six patients were selected by non-probability convenience sampling technique and randomly divided into either neuromuscular training or strength training group. Sample included 20-40 years aged adults with unilateral anterior cruciate ligament injury who had undergone surgical reconstruction of ACL two months ago using hamstring graft. Patients were assessed using the Cincinnati Knee Score for function, Numeric Pain Rating Scale (NPRS) for pain, SF-36 for quality of life, and Single Leg Hop, Triple Hop, Crossover Hop and 6-meter Hop test for power and strength. Data was analyzed using SPSS Version-21. A t-test was used to assess difference between groups. P<0.05 was considered significant.

**Results:**

Results revealed that neuromuscular training is statistically significant in reducing pain (p<0.001) and improving function (p<0.001), power & strength (p<0.001) and quality of life (p=0.001).

**Conclusion:**

Study concludes that compared to strength training, neuromuscular training was significantly more effective in reducing pain; improving function, quality of life, strength and power.

## INTRODUCTION

Problems and injuries of the knee joint are on the rise due to an increase in incidence of injuries in daily life including road traffic accidents (RTA) and industrial accidents.^1^ Ligamentous injuries being the commonest injuries of knee among which Anterior Cruciate Ligament (ACL) being most commonly injured especially when knees go into valgus position.^2^ ACL injuries involve other ligaments too including medical collateral ligament and menisci involving valgus movement’s compression is always on lateral side.^3^

The incidence of ACL injuries in United States alone is reported to be 1 in 3500 per year with higher risk in females^2^ and 300,000 ACL reconstructive surgeries performed each ear.^4^ However prevalence studies for this part of the globe are lacking.^5^ Traumatic injuries in metropolitan city of Karachi are on the rise with trauma on road being commonest (61.3%) injuries in one study,^6^ however no mention of ACL injury was noted.

Reconstructive surgical repair of ACL and Anterolateral ligament (ALL) with tenodesis results in improved knee stability in cases of ACL with anteriolateral (AL) rotational instability.^7^ However a systematic review revealed no difference between conservative treatment (rehabilitation) group and surgery group of cases with acute injury of ACL, though those having unstable knee following ACL rupture did require surgery at a later stage.^8^

Neuromuscular control is essentially required to initiate a movement, and to meet this end the nervous and muscular system work together with each other. This control is important in efficacy of a movement. Some rehabilitation exercises train the neuromuscular components of movement. It is important aspect in ACL rehab training because it plays a significant role in ACL injuries. It is significant to understand the biomechanics of how to stand after jumping and after knowing all precautionary measures this risk could be reduced. This training program includes plyometric training, balance training and some rehabilitation training. Literature has also demonstrated NMT’s protective role.^9^ Similarly, neuromuscular control plays important role to restore and improve the function of joints and body condition after injury. Hence, long term measures should be focused including movement patterns, coordination, muscle strength, endurance, and agility to prevent further injury and recurrence.^10^

Strength training is also an important research approved part of ACL rehabilitation. A number of exercise plans are devised to rehabilitate a patient after ACL reconstruction but the optimal time for incorporating different exercises in plan should be the utmost priority. After relieving pain and swelling, the major focus of rehabilitation is to target knee extensors and related muscles and for this purpose regular strength training is a viable option. This enhances the neuromuscular function of knee extensors. An injured athlete is required to go for restoration of quadriceps strength and for this strength and conditioning plans can be incorporated. These plans will help in improving muscle mass and high performance strength. Consultants can face challenges interacting with such patients i.e., facilitating muscle-tendon and neural adaptations whilst being careful of health constraints and safety of athlete.^11^

With advancing research, rehabilitation concepts of ACLR are continuously changing from time to time. An RCT to compare NT and ST revealed that at six months post training there was significant benefit noted with Cincinnati Knee Score and Visual Analogue Scale (VAS), however no significant difference was observed in hop, proprioception, tests of muscular strength and balance.^12^

The literature shows that a number of exercise plans are effective to rehabilitate ACL injuries. But a very few studies focused on neuromuscular training and its comparison with strength training in the long term follow up after ACL reconstruction.^12^ Hence keeping in view dearth of literature on the subject and rising traumatic injuries in the local context.^6^ with ACL injury associated with sports being commonest (32%) followed by road side accidents (21%),^13^ this study was conceived with the objective to determine the effectiveness of neuromuscular physical therapy as compared to strength training following anterior cruciate ligament reconstruction in terms of pain, function, quality of life, strength and power of participants. This study is of significant importance since it will help better manage the rising knee trauma cases and act as a valuable addition to literature from this part of the globe and act as a research base for future studies.

## METHODS

This single-blinded, randomized controlled trial was conducted over a period of six months from 1^st^ July 2020 and 31^st^ December 2020 after obtaining ethical approval of study protocol from Research Ethical Committee of the Riphah International University, Islamabad with Ref # Riphah/RCRS/REC/00587 and RCT subsequently registered with U.S. National Library of Medicine Clinical Trials.gov Identifier No: NCT04355078. A sample of N=76 participants was recruited using non-probability convenient sampling from Kanaan Physiotherapy & Spine Rehabilitation clinic. Sample size was calculated using Open Epi online sample size calculator.^14^ With evidence of higher prevalence in 20-30 years age group,^13^ sample population in current study included both genders, aged 20-40 years with unilateral ACL injury and had undergone surgical reconstruction of ACL by one surgeon, two months ago using hamstring graft, received physical therapy right after surgery to reduce swelling and to gain ROM. Cases with history of previous complicated knee surgery; non-operative treatment, partial ACL tear, bilateral ACL injury, associated ligament pathology that required surgical intervention at the time of the index surgery, outer bridge grade III or IV chondral injury, revision ACL reconstruction; complex associated injuries; recent re-injured in one month & those with other diseases like cancer, arthritis, bleeding disorders, organic referred pain, pregnancy etc. were excluded from the study.

For data collection tool utilized included basic demographic sheet including patients’ age, gender, body weight and height, Cincinnati Knee Score for function, Numeric Pain Rating Scale (NPRS) for pain, and English version of 36-Item Short-Form Health Survey (SF-36) for quality of life and single leg, triple, cross over and 6-meter hop test for assessing the power and strength.^15^ All participants were able to perform the tests.

Study was initiated after obtaining written informed consent of participants. A through history and physical examination including assessment of the knee was done by the researcher at the first visit, followed by assessment of function utilizing the Cincinnati Knee Score, pain using Numeric Pain Rating Scale (NPRS), and QoL using 36-Item Short-Form Health Survey (SF-36) and, Single leg Hop, Triple Hop, Crossover Hop and 6-meter Hop test for power and strength.^15^ SF-36 developed by RAND was utilized following acceptance of terms and conditions of SF-36 usage by all authors.

Randomization of sample was done into two groups using envelope method and even number patients were allotted Group-1 (Neuromuscular Training group), and odd number to Group-2 (Strength Training Group). Exercise protocol for Neuromuscular Training Group comprised:

1. Week one and two: one set each of Treadmill walking five minutes/day; exercises including squatting, single leg stance, balance reach leg and arm 10 repetitions (reps) each; lunge exercises five repetitions bilaterally; step up and down exercise 10 resp; single leg standing on balance mat 10 reps with 10 sec hold; back and side walking five steps each side, one and two leg wobble board exercise 1 min each.
2. Week three and four: One set each of Lunge exercise 10 reps; single leg stance, trampoline throwing ball in different directions five times each side; step-up wobble board 10 reps; single board exercise, and single leg stance with weights eyes closed five minutes with 10 sec hold; wobble board standing eyes closed five times with 10 seconds hold, squatting exercises, wobble board 10 reps, 10 Jumps with two legs on trampoline.
3. Week Five and Six: Running on treadmill and trampoline five minutes/ day; jump training on trampoline with increased knee flexion 10 reps; 180-degree jump on trampoline 10 jumps; running backwards, jump up and down from a step 10 steps each; running figure of 8, stop-tum-run and agility drills with slow speed five minutes each.

***Strength Training Group Intervention protocol included:***

1. Week One and Two: Three sets of exercises with Straight leg raising, supine position isometric quadriceps contraction, supine position- knee flexion and extension ROM exercises with heal in contact with bench; prone position straight leg raising; knee flexion and extension ROM; stationary biking before reaching 100 degree of flexion; standing-full weight bearing, controlled balance double limb support during parallel and diagonal stance, controlled knee extension, emphasis on full knee extension; standing heal rising exercises; one and two leg wobble board exercise; step up and down low height; squatting exercises without bars; hamstrings, hip adductor and abductor strengthening exercises 10 reps each.
2. Week Three and Four: Three sets of exercises each including Single leg heel rising; step up and down; squatting with bars; hip abduction/ adduction; hamstrings training in prone and sitting; lunges, anterior and lateral; leg press; single leg stance balance; running on treadmill five minutes 10 reps each.
3. Week Five and Six. Same exercises as Week Three and Four with increased load. three sets of 12 to 15 reps.

Intervention was done according to allotted group by physical therapist. All participants received a total of 18 treatment sessions over a 6-week period (three sessions per week). Participants were reassessed by an independent assessor after completing 18 sessions (post treatment readings).

For data entry and analysis IBM statistical package of Social Sciences Version 21 was used. Descriptive statistics was utilized. Quantitative variables were presented as means±SD. Kolmogorov-Smirnov test was to check the normality of the data. A Pre and post intervention comparison was done using parametric methods as data was normally distributed. For within group comparison Paired sample t-test and for between groups comparison independent t test was applied. P<0.05 was considered significant.

## RESULTS

Current study sample n=76 with no dropouts revealed (Table-I) a mean age of 31.13 ± 5.03 years in Neuromuscular Training Group and 29.92 ±5.71 for Strength Training Group with more cases (n=40) in the 31-40 years age group with no significant difference between the groups. Majority of population (n=43) were males and most (n=29) were in the 18.5-<25 BMI group. There was no significant difference as regards gender and BMI between the treatment groups.

**Table-I:** Demographic characteristics of Sample population (N=76).

Variable		Training Group		Chi-Square

Group	Category	Neuromuscular (n)	Strength (n)	X2, P-Value
Age Group (Years)	20-30 (36)	17	19	0.21,.819
	31-40 (40)	21	19	
	Mean±SD	31.13 ± 5.03	29.92 ± 5.71	
Gender	Male (43)	18	25	2.62,.165
	Female (33)	20	13	
BMI Category	<18.5 (21)	11	10	2.24,.523
	18.5 - <25 (29)	12	17	
	25 - <30 (19)	10	9	
	>30 (7)	5	2	

T-test statistics (Table-II) between the Neuromuscular training group and Strength training group revealed significant (p<0.001) difference between Post treatment NPRS values with decreased in post treatment Neuromuscular training group with mean value 1.58 ±1.45 vs 4.21±1.12 for strength training group Similarly the mean Cincinnati Knee System Score in Neuromuscular Training Group was 374.21 ±28.44 vs 346.05± 36.87 in Strength Training Group which was clinically significant (p<0.001) in NMT group. SF-36 scores revealed significant (P=0.002) difference in across the 02 groups with higher scores for NMT 76.05±10.34. The post intervention results of OLH Test, TH Test, CH Test and 6-MH test were also significant with p=0.029, P<0.001, p<0.001, &p<0.001 respectively with better scores for NMT group.

**Table-II:** Test/ Timing Versus Training Group Cross Tabulation. Independent sample T-Test Statistics (N=76).

Test	Timing	Training Group		

		Neuromuscular Training (38)	Strength Training (38)	T-test

		Mean±SD	Mean±SD	t,p-value
NPRS	Pre-Intervention	6.76±1.344	6.63±1.282	.437,.664
	Post-Intervention	1.58±1.445	4.21±1.119	-8.87,.000
Cincinnati Knee System Score	Pre-Intervention	206.05±46.935	227.63±56.302	-1.81,.074
	Post-Intervention	374.21±28.44	346.05±36.874	3.73,.000
SF-36	Pre-Intervention	27.5±12.234	31.71±13.719	-1.41,.162
	Post-Intervention	76.05±10.343	68.29±10.415	3.26,.002
One Leg Hop (cm)	Pre-Intervention	119.84±11.535	121.29±8.69	-618,.539
	Post-Intervention	155.53±8.67	150.97±9.155	2.22,.029
Triple Hop (cm)	Pre-Intervention	433.82±10.353	429.76±5.528	2.12,.037
	Post-Intervention	477.92±12.16	468.68±7.847	3.93,.000
Cross Over Hop (cm)	Pre-Intervention	386.66±14.751	389.03±13.369	-.733,.466
	Post-Intervention	450±9.34	430.66±8.83	9.27,.000
6-Meter Hop (seconds)	Pre-Intervention	1.81±0.101	1.8±0.08	.076,.940
	Post-Intervention	1.64±0.054	1.68±0.05	-3.65,.000

Results of paired sample t-test statistics (Table-III) to compare pre-intervention and post-intervention values within NMT & ST groups revealed significant (p<0.001) improvement in post intervention scores for NPRS, Cincinnati knee score, SF-36, OLH, TH, CH, 6-MH in both groups with Neuromuscular Training group having comparatively better effects.

**Table-III:** Training Group / Test Versus Intervention Time Cross Tabulation. Paired sample T-Test Statistics (N=76).

Training Group	Test	Timing	

		Pre-Intervention	Post-Intervention	t, p-value

		Mean±SD	Mean±SD	
Neuromuscular Training	NPRS	6.76±1.344	1.58±1.445	19.46,.000
	Cincinnati Knee System Score	206.05±46.935	374.21±28.44	-150.85,.000
	SF36	27.5±12.234	76.05±10.343	-42.82,.000
	One Leg Hop (cm)	119.84±11.535	155.53±8.67	-33.60,.000
	Triple Hop (cm)	433.82±10.353	477.92±12.16	-41.67,.000
	Cross Over Hop (cm)	386.66±14.751	450±9.34	-59.88,.000
	6-Meter Hop (seconds)	1.81±0.101	1.64±0.054	15.27,.000
Strength Training	NPRS	6.76±1.344	1.58±1.445	15.74,.000
	Cincinnati Knee System Score	206.05±46.935	374.21±28.44	-12.03,.000
	SF36	27.5±12.234	76.05±10.343	-12.00.000
	One Leg Hop (cm)	119.84±11.535	155.53±8.67	-29.93,.000
	Triple Hop (cm)	433.82±10.353	477.92±12.16	-39.44,.000
	Cross Over Hop (cm)	386.66±14.751	450±9.34	-32.93,.000
	6-Meter Hop (seconds)	1.81±0.101	1.64±0.054	14.55,.000

## DISCUSSION

Neuromuscular training was an effective intervention in improving all elements associated with ACL injury including pain and strength and function.^15^ Also Neuromuscular training (NMT) was found to be effective in ACL rehabilitation with significant improvement in global knee functions as depicted by the Cincinnati Knee Scores and Visual Analogue Scale scores compared to Strength training, however no significant difference was observed among the groups for hop, muscle strength, proprioception and balance.^12^ Similarly current study also compared two non-invasive treatment techniques, one was NMT and other was strength training to see their effectiveness on pain reduction, power, strength and functional mobility in post-operative ACL rehabilitation. Statistically significant difference with P<0.001 was noted in results of NPRS in between group analysis with significant decrease in pain in NMT group (1.58 ±1.45) compared to ST group (4.21±1.12). A systematic review of 32 studies conducted by Van Grinsven revealed similar results as far as pain is concerned with an accelerated protocol excluding post-surgery bracing.^16^

Neuromuscular training to improve the deficit improves strength and reduce the chances of re-injury thus preparing a person to reach optimum functional levels.^17^ Results of current study revealed significant difference with p<0.001 between Post-treatment Cincinnati Score values of 02 groups. These results were supported by another RCT in which neuromuscular training was compared to traditional strength training. Both rehabilitation programs lasted for six months after ACL surgery. There was significant improvement in Cincinnati Knee Scores and visual analog scale (VAS) scores at six months follow up in the NMT group as compared to the ST group.^12^ Results were further supported by an experimental study in which it was found that perturbation training was found to very effective in improving knee function.^18^

Being a suitable tool to assess the quality of life in assessing impact of treatment of injury SF 36 was used to assess the difference in the treatment of ACL injury^19^ in current study which revealed statistically significant difference between Post-treatment SF 36 score values of 02 groups with p=0.002 with higher increase in scores following NMT (76.05 ±10.343) compared to ST group (68.29 ± 10.42). These results are consistent with another previous research which shows the similar results in terms of improvement in quality of life measured by SF-36.^12^

Current study also revealed significant (p=0.029) difference between Post-Treatment One Leg Hop Score values of 02 groups with higher scores in post treatment NMT group (155.53 ± 8.67) compared to ST group (150.97 ± 9.15). These results are consistent with previous research which shows improvement in one leg Hop test after NMT which resulted in quantitative improvement in measurements.^20^ This is also in line with general studies involving NTM including a study on soccer players in which short term use of NMT resulted in improved one leg hop test results.^21^

Present study also revealed significant difference with p<0.001 between Post-Treatment Triple Hop Score values of 02 groups with higher increase in scores of NMT group (477.92 ± 12.16) compared to ST group (468.68 ± 7.85). An RCT revealed similar results with higher scores for both triple jump test and one leg hop test.^12^

Similarly, current study revealed significant difference with p<0.001 between Post-Treatment Cross Over Hop Score values of 02 groups with higher increase in scores of post treatment of NMT group (450± 9.34) compared to ST group (430.66 ± 8.83). The results also showed that there was statistically significant (p<0.001) difference between Post-Treatment 6-meter Hop Score values of 02 groups with greater decrease in time in post treatment of NMT group (1.64± 0.05) compared to ST group (1.68 ± 0.05). Previous literature shows similar results with more clinically significant improvement in NMT group in terms of crossover hop test and 6-meter hop test with intervention provided within 3 months of ACL injury 22 in one study and with pretest within four to seven months in another study.^20^

Hence the current study shows statistically as well as clinically significant results in terms of Cincinnati Knee score, SF-36, One leg hop test, triple hop test, crossover hop test and 6-meter hop test, which are consistent with literature.^20^

### Limitations of the study:

Most of population belonged to male gender, hence results cannot be generalized to entire population. Also, no long term follow-up was conducted.

## CONCLUSIONS

Study concludes that compared to strength training, neuromuscular training was significantly (p<0.05) more effective in reducing pain; improving function, quality of life, strength and power.

### Authors Contribution:

**KK** did the data collection, analysis, interpretation, responsible for accuracy and integrity of the research.

**NA** Prepared the manuscript

**GS** designed & critically revised the article.

**FA**. Conceived and did the methodology & Literature Review.
